# A study on the construction of a nurse refresher training system in traditional Chinese medicine hospitals using the Delphi method

**DOI:** 10.3389/fpubh.2025.1618002

**Published:** 2025-07-23

**Authors:** Ying An, Huirong Xu, Qiuxiao Ma, Xiujun Wang, Ying Ding, Ruxin Yang, Songbai Yang

**Affiliations:** ^1^Spinal Surgery Department I, Wangjing Hospital of CACMS, Beijing, China; ^2^Nursing Department, Wangjing Hospital of CACMS, Beijing, China; ^3^Department of Cardiovascular, Wangjing Hospital of CACMS, Beijing, China; ^4^Orthopedic and Joint Department I, Wangjing Hospital of CACMS, Beijing, China; ^5^Materials Department, Wangjing Hospital of CACMS, Beijing, China; ^6^Sports Medicine Department I Ward and Vascular Surgery Ward, Wangjing Hospital of CACMS, Beijing, China; ^7^Emergency Department, Wangjing Hospital of CACMS, Beijing, China

**Keywords:** traditional Chinese medicine nursing, traditional Chinese medicine nursing staff, training system, Delphi method, health education

## Abstract

**Background:**

This study aims to develop a standardized advanced training system for nursing staff in traditional Chinese medicine (TCM) hospitals and verify its effectiveness.

**Methods:**

Based on literature review, training demand research, and semi-structured interviews, this study established a draft training system for TCM nursing staff. 12 experts refined the system through two rounds of correspondence using the Delphi method. Forty-five teachers engaged in clinical teaching were invited to evaluate the course quality, course setting, training programs, training effectiveness, teacher teaching quality, and full-score evaluation rate before and after the application of the training system.

**Results:**

The recovery rates for the two rounds of questionnaires were 100 and 91.67%, respectively, with authority coefficients of 0.938 and 0.953, and coordination coefficients of 0.233 and 0.239. The final training system included 15 first-level, 49 s-level, and 85 third-level indexes. Scores in the training effect evaluation significantly improved after applying the training system (*p* < 0.001).

**Conclusion:**

It is concluded that the training system constructed in this study is scientific, reliable, and valid, and can provide guidance for the training of TCM nursing staff.

## 1 Introduction

Traditional Chinese medicine (TCM) nursing is a significant discipline within the field of TCM, guided by TCM theory. It employs a holistic concept of evidence-based care and utilizes distinctive TCM characteristic nursing techniques. By integrating modern nursing theory and technology, TCM nursing aims to deliver comprehensive and personalized care to patients (1). In recent years, with changes in the medical environment and the continuous improvement of people’s health needs, the theory and technology of TCM nursing have ushered in new development opportunities (2). At present, TCM nursing is in a period of significant development of the discipline in China, and the “China Nursing Career Development Plan (2021–2025)” underscores the imperative to nurture TCM nursing talent proactively, advance the establishment of the TCM nursing discipline, and optimally utilize the function of TCM nursing in the treatment of diseases, management of chronic illnesses, health care, rehabilitation, and promotion of health and aging, among other domains (3). However, higher education in TCM nursing began relatively recently, and there is a lack of practical and scientific research talent in this field (4). Additionally, the nursing staff of TCM hospitals are primarily graduates from Western medical nursing schools, and the professional knowledge of nurses in TCM is lacking. Their mastery of TCM technology is limited, and their mastery in nursing techniques that require high specialization and strong emphasis on safety is unsatisfactory, which leads to inconsistencies in the levels of TCM nursing knowledge and skills among the nursing staff of TCM hospitals, making it difficult for them to meet the actual needs of clinical work (2). Therefore, it is essential to develop scientific, systematic, and standardized refresher training programs for TCM hospital nursing staff. This study used the Delphi expert correspondence method to construct a set of standardized nursing staff refresher training systems in TCM hospitals, and evaluated its application effect, aiming to optimize the content of TCM nursing training, improving the professional quality and skills of TCM nursing staff, providing a reference basis for the community and TCM hospitals at all levels to carry out TCM nursing personnel training.

## 2 Materials and methods

### 2.1 Formation of research team

The research team consisted of 10 members, including a chief physician, a chief nurse, a deputy chief nurse, five charge nurses, a nurse practitioner, and a senior economist. Each team member had specific responsibilities, including conducting a literature review and analysis, preparing an expert correspondence questionnaire, selecting experts, distributing and collecting correspondence questionnaires, and compiling recommendations and statistical analyses of the data. The study was approved by the Medical Ethics Committee of our hospital (Ethics approval no. WJEC-KT-2022-025-P001). In Delphi research, we adhere strictly to ethical research standards to ensure the rights and interests of all participating experts are protected. After fully understanding the relevant information about the study, the experts voluntarily decided whether to participate and signed informed consent forms. Throughout the entire research process, we kept all expert data and opinions strictly confidential and used them solely for research purposes. We also employed anonymous processing methods to protect the experts’ privacy.

### 2.2 Draft framework for the preliminary construction of a system of advanced training for nursing staff in TCM hospitals

In the early stages of this study, a preliminary draft of the training system was formed based on literature reviews, research into training needs, expert interviews, and clinical practice experience. The process is divided into four major steps: (1) Literature review of domestic and international research on TCM nursing training and evaluation, summarizing the hot spots of research and application; (2) Conduct questionnaire surveys to assess the training needs of nursing staff in three Chinese medicine hospitals to clarify the training courses and skills development requirements of clinical Chinese medicine nursing staff; (3) Semi-structured interviews were conducted with 37 nursing experts to explore the training objectives, curriculum, core competencies, training methods, and training hours of nursing staff in TCM hospitals. Members of the research team organized the interviews into written texts within 48 h of their completion; (4) Combining the literature review, training needs research, expert interview results, and experience in clinical training, the group was convened to discuss and initially outline the contents of a preliminary draft for a refresher training system for nursing staff in Chinese medicine hospitals.

### 2.3 Preparation of expert consultation questionnaire

Prepare an expert questionnaire based on the draft of the training system. The questionnaire should consist of four parts: (1) questionnaire guidance: introduce the background, purpose, significance, and notes on filling out the form of this study; (2) expert information questionnaire: include the expert’s age, gender, years of work experience, education, title, and field of specialization; (3) preliminary draft of the TCM hospital nursing staff refresher training system project: use the Likert 5-level scale to rate the importance of each indicator, with unimportant, not too important, generally important, important, and very important scoring 1, 2, 3, 4, and 5 points, respectively, and adding an expert modification column and a supplemental column after each level of entry (Supplementary Table 1) (5); (4) the expert familiarity questionnaire and the expert judgment basis questionnaire: Among them, the expert familiarity questionnaire is divided into 5 levels according to very familiar, more familiar, generally familiar, less familiar, and very unfamiliar, and is assigned 1.0, 0.75, 0.5, 0.25, and 0 points, respectively. The expert judgment questionnaire includes four aspects: theoretical analysis, practical experience, understanding of domestic and foreign counterparts, and intuitive feeling. Each aspect is divided into three levels: large, medium, and small. The three levels of “theoretical analysis” are assigned 0.3, 0.2, and 0.1 points, respectively. “Practical experience” is assigned 0.3, 0.2, and 0.1 points, respectively. The three levels of “intuitive feeling” and “peer understanding” are assigned 0.1 points.

### 2.4 Selection of experts

This research was conducted in two rounds of expert consultations from April 2023 to September 2023. The inclusion criteria for experts were as follows: (1) Over 10 years of experience in Chinese medicine nursing, integrated Chinese and Western medicine nursing, Chinese medicine nursing education, or Chinese medicine nursing management; (2) Bachelor’s degree or above; (3) Intermediate title or above; (4) Voluntary participation in this study, and have a certain degree of enthusiasm for this study.

### 2.5 Implementation of Delphi expert consultation

During the two rounds of expert consultations, questionnaires were distributed to the experts via e-mail. Each round of consultation was completed within 2 weeks. After the first round of consultation, we revised and improved the training program based on the experts’ comments and suggestions and constructed the second round of expert questionnaires. After each round of expert consultation, we established the following selection criteria for each indicator (6): (1) A mean importance score >3.5. As a critical value, 3.5 is in the upper middle level, which means that experts generally believe that this index has high importance, can significantly affect the research object or result, and is worth retaining for subsequent research or practice; (2) Coefficient of variation <0.25 for each indicator; and (3) frequency of full score > 20% for each indicator. Indicators that met all three criteria were included, while those that did not meet any of the criteria were excluded. When there is a divergence of expert opinions, an anonymous online discussion is organized to conduct in-depth explorations that integrate classical theories of TCM with evidence from modern clinical research, aiming to reach a consensus or seek a compromise.

### 2.6 Evaluation of the effectiveness of the application of the training system

The training system for the advanced training of nursing staff in Chinese medicine hospitals, as determined by expert correspondence, was piloted in three hospitals simultaneously. Forty-five teachers (including four head nurses and forty-one heads of teaching teams) were invited to rate the training effect before and after the implementation of the training system. The evaluation included five aspects: course quality, course design, training program, training effectiveness, and teacher quality evaluation. Each aspect’s evaluation standard is divided into five levels: poor, unsatisfactory, general, satisfactory, and very satisfactory. Values of 1, 2, 3, 4, and 5 are assigned to each level, respectively.

### 2.7 Statistical analysis methods

Data collection and statistical analysis were performed using Excel 2016 and IBM SPSS Statistics 27.0 software. Measurement data were described as mean ± standard deviation (*x-*±*s*), and count data were described using frequency and percentage. The authority and reliability of the results were determined by calculating the expert positivity coefficient, the degree of authority, the degree of concentration of opinions, and the degree of coordination. The expert positivity coefficient reflects the response rate to the expert’s advisory letter, with a higher response rate indicating a higher level of expert positivity. The degree of expert authority is determined by the experts themselves and is expressed by the coefficient of expert authority (Cr), which is calculated using the expert judgment coefficient (Ca) and the expert familiarity coefficient (Cs): Cr = (Ca + Cs)/2. The degree of concentration of expert opinions is expressed by the mean, coefficient of variation, and frequency of full scores. The degree of harmonization of expert opinions was expressed by the Kendall W coefficient. Scores before and after training were compared using a paired samples t-test. A *p* value < 0.05 indicates a statistically significant difference.

## 3 Results

### 3.1 General information on the experts

A total of 12 experts were consulted in the first round of this study, including two males and 10 females. Among the experts, six were nursing administrators, three were medical researchers, one was involved in TCM nursing, and two were engaged in integrated TCM nursing. All participants were experienced in their respective fields. The educational background of the experts varied, with eight holding bachelor’s degrees, two holding master’s degrees, and two holding doctorate degrees. In terms of titles, eight held senior titles, three held deputy senior titles, and one held an intermediate title (Tables 1, 2).

**Table 1:** The general information on the experts consulted in the first round (*n* = 12).

General information	Classification	Number	Percentage (%)
Gender	Female	10	83.33
	Male	2	16.67
Age (years)	30–39	3	25.00
	40–49	3	25.00
	50–59	5	41.67
	≥60	1	8.33
Working experience (years)	10–19	4	33.33
	20–29	2	16.67
	≥30	6	50
Education background	Bachelor’s degree	8	66.67
	Master’s degree	2	16.67
	Doctorate	2	16.67
Specialization field	Nursing management	6	50.00
	Medical care	3	25.00
	Traditional Chinese Medicine nursing	1	8.33
	Combined traditional Chinese and Western medicine nursing	2	16.67
Professional title	Intermediate title	1	8.33
	Deputy senior title	3	25
	Senior title	8	66.67
Familiarity with the contents of the study	Very familiar	8	66.67
	More familiar	4	33.33
	Generally familiar	0	0.00
	Less familiar	0	0.00
	Very unfamiliar	0	0.00

**Table 2:** The general information on the experts consulted in the second round (*n* = 11).

General information	Classification	Number	Percentage (%)
Gender	Female	9	81.82
	Male	2	18.18
Age (years)	30–39	2	18.18
	40–49	3	27.27
	50–59	5	45.45
	≥60	1	9.09
Working experience (years)	10–19	3	27.27
	20–29	1	9.09
	≥30	7	63.64
Education background	Bachelor’s degree	7	63.64
	Master’s degree	2	18.18
	Doctorate	2	18.18
Specialization field	Nursing management	6	54.55
	Medical care	3	27.27
	Traditional Chinese Medicine nursing	1	9.09
	Combined traditional Chinese and Western medicine nursing	1	9.09
Professional title	Intermediate title	0	8.33
	Deputy senior title	3	27.27
	Senior title	8	66.67
Graduate student mentor	Yes	8	72.73
	No	3	27.27
Familiarity with the contents of the study	Very familiar	8	72.73
	More familiar	3	27.27
	Generally familiar	0	0.00
	Less familiar	0	0.00
	Very unfamiliar	0	0.00

### 3.2 Expert positive degree and authority coefficient

The recovery rates for the two rounds of questionnaires were 100 and 91.67%, respectively. In the first round, nine experts proposed modifications or additional comments, while in the second round, three experts proposed further modifications. In the first round, Cr was 0.938. In the second round, Cr was 0.953 (Table 3).

**Table 3:** Degree of authority of experts.

Round	Judgment coefficient (Ca)	Familiarity coefficient (Cs)	Authority coefficient (Cr)
1	0.958	0.917	0.938
2	0.973	0.932	0.953

### 3.3 Summary of expert opinions

The modifications made to the indicators of the training system after two rounds of expert consultation are summarized below:

1. Training objectives: Two experts suggested that TCM nursing personnel should have certain innovative abilities and the ability to popularize TCM nursing. After discussion in the panel, the experts’ opinions were incorporated, and the indicators of “possessing innovative ability and being able to conduct research on TCM nursing” and “possessing the ability to popularize TCM nursing” were added.
2. Admission criteria: Some experts pointed out that nursing personnel participating in the training should have a background in TCM nursing or have systematically received training in TCM nursing knowledge or skills. After discussion in the panel and accepting the experts’ opinions, the indicator of “having a certain degree of clinical nursing experience but having not participated in training in TCM knowledge” was deleted, and the indicator of “having graduated from a TCM nursing program or having received training in TCM knowledge” was added. The indicator of “graduates of TCM nursing” or “graduates of Western medicine nursing who have participated in training courses in Western studies” was amended to “graduates of TCM nursing or graduates of Western medicine nursing who have participated in training courses in Western studies or have received systematic training in TCM nursing knowledge and skills.”
3. Training methods: Some experts suggested replacing “scenario simulation” with “workshop” in the method of teaching and “clinical inspection” with “operational room inspection.” In addition, the experts suggested that the “place of practice” indicator could be included in the “training methods” indicator. After the discussion in the group, these suggestions were accepted.
4. TCM nursing program: One expert suggested adding training in the implementation of TCM nursing programs for common pediatric conditions. After discussion in the group, it was unanimously decided to add this indicator.
5. Assessment indicators: Some experts suggested adding a form of assessment, and after discussion in the group, it was unanimously decided to replace the “case report” with a form of assessment in which any one of the “five case reports” is selected.

### 3.4 Degree of concentration and coordination of expert opinions

The first round of consultation consisted of 148 indicators, the mean range of indicator importance ratings was 3.25–4.92, the coefficient of variation ranged from 0.06–0.44, and the Kendall’s coefficient of Concordance was 0.233 (*p* < 0.05) for all indicators. The second round of consultation included 151 indicators, with an average range of importance ratings of 3.73–4.82, a coefficient of variation of 0.08–0.27, and a Kendall’s coefficient of 0.239 for all indicators (*p* < 0.05).

### 3.5 Survey statistics of expert training duration in two rounds

In the two rounds of training duration statistics, regarding theoretical training hours, the proportion of “100–150 h” increased significantly from 33.33% in the first round to 63.64% in the second round, indicating a more concentrated focus on expert opinions. For practical training hours, in the first round, “300–350 h” accounted for 50.00%, and “150–200 h” represented 33.33%; in the second round, “350–400 h” led with 36.36%, while “300–350 h” fell to 27.27%. The duration of TCM ward practice was mainly “3 months” in both rounds, with a slight decrease from 66.67% in the first round to 63.64% in the second round. As for TCM nursing outpatient practice time, it shifted from “1 month” (41.67%), “2 months” (33.33%), and “3 months” (25.00%) in the first round to “3 months” (54.55%), “1 month” (27.27%), and “2 months” (18.18%) in the second round, showing that experts favored a longer duration for outpatient practice (Tables 4, 5).

**Table 4:** Statistics on the length of the first round of training number of cases (%).

Item	Times	Count	Percentage (%)
Training theoretical hours	50–100 h	3	25.00
	100–150 h	4	33.33
	150–200 h	4	33.33
	200–250 h	1	8.33
	250–300 h	2	16.67
Training practical hours	200–250 h	6	50.00
	250–300 h	2	16.67
	300–350 h	2	16.67
	350–400 h	1	8.33
	400–450 h	8	66.67
Training duration for integrated Chinese and western medicine ward	1 month	3	25.00
	3 months	5	41.67
	6 months	4	33.33
Training duration for Chinese nursing clinic	1 month	3	25.00
	2 months	3	25.00
	3 months	4	33.33
Valid responses	12		

**Table 5:** Statistics on the length of the second round of training number of cases (%).

Item	Times	Count	Percentage (%)
Training theoretical hours	50–100 h	0	0
	100–150 h	7	63.64
	150–200 h	2	18.18
	200–250 h	1	9.09
	250–300 h	1	9.09
Training practical hours	200–250 h	1	9.09
	250–300 h	2	18.18
	300–350 h	3	27.27
	350–400 h	4	36.36
	400–450 h	1	9.09
Training duration for integrated Chinese and western medicine ward	1 month	1	9.09
	3 months	7	63.64
	6 months	3	27.27
Training duration for Chinese nursing clinic	1 month	3	27.27
	2 months	2	18.18
	3 months	6	54.55
Valid responses	11		

### 3.6 Final program of the continuing education and training system for nursing staff in TCM hospitals

After two rounds of expert consultation, expert opinions converged, and the finalized advanced training system for nursing staff in TCM hospitals included 15 primary indicators, 49 secondary indicators, and 85 tertiary indicators (Supplementary Table 2). The indicators of training objectives (4.41 ± 0.70, CV = 0.16), admission criteria (4.59 ± 0.67, CV = 0.15), training methods and specific approaches (4.41 ± 0.75, CV = 0.17), practice locations (4.73 ± 0.55, CV = 0.12), nursing professionalism (4.67 ± 0.54, CV = 0.12), laws and regulations (4.68 ± 0.48, CV = 0.10), basic TCM (4.29 ± 0.72, CV = 0.17), TCM nursing courses (4.48 ± 0.76, CV = 0.17), TCM nursing specialization plans (4.65 ± 0.56, CV = 0.12), and capability development (4.34 ± 0.64, CV = 0.15) were highly recognized by the experts.

### 3.7 Effectiveness of the application of the training system

The scores of each item in the five aspects of course quality, course setting, training program, training effectiveness, and evaluation of the quality of teaching teachers were significantly higher after the application of the training system than before (*p* < 0.001; Table 6).

**Table 6:** Comparison of training effect scores before and after the application of nursing staff refresher training system in TCM hospitals.

Evaluation content	Evaluation items	*x-*±*s*		*t*	*p*	Variation coefficient		Perfect score frequency	
		Before	After			Before	After	Before	After
Course quality evaluation	Rationalization of the curriculum structure	4.16 ± 0.88	4.84 ± 0.37	−4.778	<0.001	0.21	0.08	0.44	0.84
	Course content highlights the characteristics of Traditional Chinese Medicine	4.18 ± 0.89	4.80 ± 0.40	−4.262	<0.001	0.21	0.08	0.47	0.80
Course setting	Course objective clarity	4.13 ± 0.87	4.84 ± 0.37	−4.930	<0.001	0.21	0.08	0.42	0.84
	Course content detail	4.11 ± 0.91	4.84 ± 0.37	−4.985	<0.001	0.22	0.08	0.42	0.84
Training programs	Course plan reasonableness	4.13 ± 0.81	4.84 ± 0.42	−5.220	<0.001	0.20	0.09	0.40	0.87
	Course content consistency with plan	4.16 ± 0.90	4.87 ± 0.34	−4.951	<0.001	0.22	0.07	0.47	0.87
Training effectiveness	Professional practice ability improvement	4.18 ± 0.86	4.84 ± 0.37	−4.729	<0.001	0.21	0.08	0.44	0.84
	Nursing teaching ability improvement	4.18 ± 0.83	4.80 ± 0.40	−4.514	<0.001	0.20	0.08	0.42	0.80
	Organizational management ability improvement	4.04 ± 1.00	4.82 ± 0.39	−4.875	<0.001	0.25	0.08	0.44	0.82
	Clinical thinking ability improvement	4.13 ± 0.92	4.82 ± 0.39	−4.632	<0.001	0.22	0.08	0.47	0.82
	Nursing research ability improvement	4.04 ± 0.95	4.84 ± 0.37	−5.264	<0.001	0.24	0.08	0.42	0.84
Evaluation of the quality of teaching teachers	Teaching method	4.38 ± 0.83	4.84 ± 0.37	−3.396	<0.001	0.19	0.08	0.58	0.84
	Teaching speed	4.24 ± 0.96	4.84 ± 0.37	−3.912	<0.001	0.23	0.08	0.53	0.84
	Clarity of lecture content	4.27 ± 0.86	4.82 ± 0.44	−3.819	<0.001	0.20	0.09	0.51	0.84
	Encouraging trainees to actively participate	4.27 ± 0.86	4.82 ± 0.39	−3.907	<0.001	0.20	0.08	0.51	0.82
	Effective communication with trainees	4.31 ± 0.79	4.82 ± 0.39	−3.883	<0.001	0.18	0.08	0.51	0.82
	Preceptor qualifications and experience	4.42 ± 0.75	4.87 ± 0.34	−3.666	<0.001	0.17	0.07	0.58	0.87

## 4 Discussion

In long-term life practice, TCM nursing has developed a system of non-pharmacological therapies that can be used not only for clinical treatment of diseases but also as a health maintenance technique to be promoted among community residents (7, 8). Studies have shown that TCM nursing interventions can improve the recovery of gastrointestinal function in gastric cancer patients and uterine fibroid patients after surgery, improve the prognosis of cancer patients, alleviate postoperative pain in patients with anorectal diseases, and improve the daily living ability of older adult patients with chronic diseases (9–12). Clinical research has confirmed the effectiveness of TCM nursing, leading to an expansion of its application and a gradual increase in demand among the public (10, 12). However, there is currently a shortage of TCM nursing talents, and the professional skills of nursing staff are insufficient to meet the needs of TCM clinical nursing (2). Therefore, it is necessary to establish a standardized and comprehensive training system for TCM nursing personnel in TCM hospitals and to effectively cultivate a strong workforce of TCM nursing professionals through standardized, systematic, and professional training programs to meet the needs of TCM nursing development.

This study constructed a system of refresher training for nursing staff in TCM hospitals based on the Delphi study methodology. Delphi expert consultation is often used to construct clinical scales and evaluation indicators to enhance the rigor and systematic nature of the study (13). In the Delphi method of expert consultation, the representativeness, motivation, and authority of the experts are key to the scientific validity and reliability of the indicators of consultation Construction of a Training Content System for New Nurses in Cancer Hospital Based on Competency (14). In this study, the 12 experts cover various fields, including nursing management, medical research, and TCM nursing, with a wide distribution in terms of gender, education, and professional titles. This diverse team of experts ensures the comprehensiveness and authority of the consultation results. The validity rate of the two rounds of consultation questionnaires was 100 and 91.67%, respectively, indicating that the experts were highly motivated to participate in the study. The authority coefficients of the two rounds of consultation were 0.938 and 0.953, respectively. The authority coefficients of the experts in the two rounds were >0.70, which indicated that the experts in this study had high authority and the results of the consultation were reliable (14). The Kendall coefficients for the two rounds of expert consultation are 0.233 and 0.239, respectively, and the *p* value is less than 0.05, which is statistically significant, indicating that the experts have a high degree of recognition of all the indicators in the training system of this study. These findings demonstrate the reliability and scientific validity of the training system constructed in this study.

The nursing staff refresher training system in TCM hospitals constructed in this study covered 15 primary, 49 secondary, and 85 tertiary indicators, and the importance scores of all entries were >3.5. The importance scores of all entries were greater than 3.5. This training system clearly aims to cultivate TCM nursing professionals with a strong theoretical foundation, solid clinical skills, and a high level of humanistic literacy. This objective is similar to those of previous studies (15, 16). This training system strengthens the theoretical foundation of TCM nursing for trainers across various aspects through basic courses in Chinese medicine, foundational courses in TCM nursing, and specialized courses in TCM nursing training. Based on theoretical knowledge, the clinical practice ability and adaptability of trainers are further enhanced through the training of the TCM nursing program and nursing management program. In addition to developing personnel capable of integrating theory and practice, this study also aims to cultivate innovative thinkers who can effectively promote TCM nursing. The importance scores of these two indicators are >4, and the CV index is <0.25, which fully illustrates the importance that experts attach to the cultivation of innovative ability and science popularization ability of TCM nursing personnel. Currently, the educational level of nursing personnel in TCM hospitals is relatively low, with a noticeable lack of highly educated personnel, resulting in insufficient innovation capacity in TCM nursing theory and traditional TCM technology (4). In comparison to Western nursing research, the current state of scientific research in TCM nursing is less promising. There are fewer research projects focused on TCM nursing, and the scope of these studies is narrower. Additionally, the quantity and quality of research are significantly lower compared to Western nursing (17). Therefore, strengthening the training of TCM nursing personnel in scientific research and innovation is of great significance to enhance their comprehensive abilities and promote the high-quality development of TCM nursing specialties. The Outline of the “Healthy China 2030” (18) Plan emphasizes the importance of integrating the popularization of medical science into people’s daily lives to improve health and promote the steady and efficient development of the popularization of medical knowledge in China. Training TCM nursing personnel in science popularization strengthens doctor-patient communication and trust, enhances health literacy, and promotes the inheritance and development of TCM nursing (19). In this study, we conducted training in health science for Chinese medicine nursing staff to improve their scientific competence. In terms of training assessment methods, this study incorporates a five-case debriefing assessment in addition to traditional theory and skills assessments. This approach aims to comprehensively evaluate trainees’ analytical, decision-making, and problem-solving abilities in real-world scenarios.

In terms of training duration statistics, experts prefer a theoretical training duration of “100–150 h” while the preferred practical training duration is gradually shifting toward “350–400 h.” The duration of TCM ward practice is mainly “3 months,” and experts prefer to extend the duration of TCM nursing outpatient practice to “3 months.” This reflects the experts’ view that the cultivation of TCM nursing talents requires a solid theoretical foundation and ample practical training, especially for outpatient practice. Extending the practice duration helps students accumulate more clinical experience and improve their ability to deal with practical issues, which aligns with the practical focus of TCM nursing.

The training system created in this study was tried out in three hospitals to verify its training effectiveness, and the results showed that the scores of each item in the five aspects of course quality, curriculum, training program, training effect, and evaluation of teachers’ teaching quality were significantly higher after the application of this training system than before the application (*p* < 0.001). Additionally, the pass rate of scores in various aspects increased after the training compared to before. These results demonstrate that the training system can effectively improve the quality and effectiveness of nursing staff training in TCM hospitals. The system plays a positive role in cultivating high-quality TCM nursing talent and promoting the development of TCM nursing.

TCM nursing is deeply rooted in Chinese traditional culture, with its theoretical foundation and operational techniques closely connected to cultural concepts, facing numerous challenges in cross-cultural communication and application. Theoretically, fundamental TCM theories, such as yin-yang and the five elements, as well as meridian theory, embody unique Oriental philosophical thought. When promoted in regions with Western cultural backgrounds, understanding difficulties may arise due to cultural differences. For example, the Western nursing system emphasizes empirical research and standardized operations, lacking intuitive cognition and acceptance of concepts like the holistic view and syndrome differentiation-based nursing in TCM nursing (20, 21). In practical operations, TCM nursing techniques such as moxibustion and tuina (massage) are influenced by cultural habits and cognition in terms of manipulation, strength, and frequency, among other factors, requiring adaptive adjustments in different cultural environments to ensure effective implementation. However, with the growing international influence of traditional Chinese medicine, it also brings opportunities for the cross-cultural application of TCM nursing training systems. In some Asian cultural circle countries, due to cultural homology, the acceptance of TCM nursing concepts and techniques is relatively high, which can be prioritized as pilot promotion areas to verify the cross-cultural applicability of the training system and optimize it based on feedback. Additionally, by integrating TCM nursing with modern evidence-based nursing and using international research methods and standards to verify TCM nursing techniques, their recognition in the global nursing field can be enhanced, laying a foundation for the cross-cultural communication of the training system.

However, this study also has certain limitations. The research only selected 12 experts for consultation, and the sample size is relatively small, which may not fully cover the views and needs of all relevant fields. In future studies, the scope of expert samples can be further expanded, and more experts from different regions and levels can be included to enhance the training system. Additionally, the long-term effects of the training system still need to be tested through broader and longer-term practice in more traditional Chinese medicine hospitals. By continuously summarizing experience and continuously optimizing the system content, it can better meet the needs of talent training for the development of traditional Chinese medicine nursing. At the same time, regarding the issue of cross-cultural applicability, more international cooperative research should be carried out to explore traditional Chinese medicine nursing training models suitable for different cultural backgrounds.

## 5 Conclusion

The nursing staff refresher training system of TCM hospitals constructed in this study is in line with the current situation and needs of TCM nursing development. The study results are both scientific and reliable, and the established index system provides guidance for the standardized training of TCM nursing staff.

## Data Availability

The raw data supporting the conclusions of this article will be made available by the authors, without undue reservation.
