# Supplementary material for: A study on the construction of a nurse refresher training system in traditional Chinese medicine hospitals using the Delphi method
Source: Front Public Health. 2025 Jul 23;13:1618002. doi: 10.3389/fpubh.2025.1618002 (PMC12325172; doi:10.3389/fpubh.2025.1618002)
Supplement: Supplementary file 1 [file Table_1.docx]

Supplementary Table 1 Likert scale of importance of lead teacher training programs [*n* (%)]

| Training Courses | | Importance rating (*n*=267) | | | | | |
| --- | --- | --- | --- | --- | --- | --- | --- |
|  |  | Very Unnecessary | Unnecessary | Average | Necessary | Very Necessary | Score (*x̅*±*s* ) |
| TCM Basic Theory | Yin-Yang Theory | 4(1.50) | 3(1.12) | 44(16.48) | 64(23.97) | 152(56.93) | 4.34±0.90 |
|  | TCM Basic Theory | 5(1.87) | 4(1.50) | 39(14.61) | 73(27.34) | 146(54.68) | 4.31±0.91 |
|  | Five Elements Theory | 7(2.62) | 3(1.12) | 43(16.10) | 75(28.09) | 139(52.06) | 4.26±0.95 |
|  | Tibetan Medical Theory | 7(2.62) | 4(1.50) | 46(17.23) | 66(24.72) | 144(53.93) | 4.26±0.97 |
|  | Essence and Qi Theory | 6(2.25) | 4(1.50) | 35(13.11) | 73(27.34) | 149(55.81) | 4.33±0.92 |
|  | Etiology and Pathogenesis | 4(1.50) | 3(1.12) | 38(14.23) | 68(25.47) | 154(57.68) | 4.37±0.88 |
| Diagnostics of Traditional Chinese Medicine | Inspection, Listening, Asking, and Palpation Methods | 5(1.87) | 7(2.62) | 31(11.61) | 67(25.09) | 157(58.80) | 4.36±0.92 |
|  | Eight-Principle Syndrome Differentiation | 6(2.25) | 8(3.00) | 42(15.73) | 64(23.97) | 147(55.06) | 4.27±0.98 |
|  | Zang - Fu Organ Syndrome Differentiation | 6(2.25) | 8(3.00) | 39(14.61) | 68.0(25.47) | 146.0(54.68) | 4.27±0.97 |
|  | Syndrome Differentiation of Pathological Nature | 7(2.62) | 8(3.00) | 39(14.61) | 71.0(26.59) | 142.0(53.18) | 4.25±0.99 |
|  | Wei-Qi-Ying-Xue Syndrome Differentiation | 7(2.62) | 7(2.62) | 42(15.73) | 68.0(25.47) | 143.0(53.56) | 4.25±0.99 |
| Traditional Chinese Medicine Health Preservation Science | Basic Principles of Health Preservation and Common Health Preservation Methods | 5(1.87) | 3(1.12) | 43(16.10) | 62.0(23.22) | 154.0(57.68) | 4.34±0.92 |
|  | Health Preservation and Healthcare Methods | 7(2.62) | 3(1.12) | 38(14.23) | 67.0(25.09) | 152.0(56.93) | 4.33±0.94 |
| Fundamentals of Traditional Chinese Medicine Nursing | Development History of Traditional Chinese Medicine Nursing | 1(0.37) | 8(3.00) | 56(20.97) | 67.0(25.09) | 135.0(50.56) | 4.22±0.91 |
|  | Basic Characteristics of Traditional Chinese Medicine Nursing | 0(0) | 6(2.25) | 46(17.23) | 72.0(26.97) | 143.0(53.56) | 4.32±0.84 |
|  | General Nursing | 1(0.37) | 5(1.87) | 36(13.48) | 68.0(25.47) | 157(58.80) | 4.40±0.82 |
|  | Syndrome - Differentiated Nursing | 0(0) | 6(2.25) | 33(12.36) | 69.0(25.84) | 159(59.55) | 4.43±0.79 |
| Traditional Chinese Medicine Dietotherapy | Basic Theories of Traditional Chinese Medicine Dietotherapy | 5(1.87) | 7(2.62) | 42(15.73) | 69(25.84) | 144(53.93) | 4.27±0.95 |
|  | Preparation of Common Dietotherapy Prescriptions | 5(1.87) | 7(2.62) | 40(14.98) | 76(28.46) | 139(52.06) | 4.26±0.94 |
| Meridians and Acupoints | Concept and Composition of Meridians | 6(2.25) | 7(2.62) | 44(16.48) | 67(25.09) | 143(53.56) | 4.25±0.97 |
|  | Names, Distribution and Course of the Twelve Regular Meridians | 7(2.62) | 7(2.62) | 47(17.60) | 61(22.85) | 145(54.31) | 4.24±1.00 |
|  | Concept and Physiological Functions of the Eight Extraordinary Meridians | 6(2.25) | 7(2.62) | 47(17.60) | 70(26.22) | 137(51.31) | 4.22±0.98 |
|  | Basic Knowledge of Acupoints | 7(2.62) | 7(2.62) | 43(16.10) | 67(25.09) | 143(53.56) | 4.24±0.99 |
|  | Syndrome Differentiation and Acupoint Selection for Common Conditions | 5(1.87) | 4(1.50) | 40(14.98) | 68(25.47) | 150(56.18) | 4.33±0.91 |
| TCM Skills | Scraping Technique | 6(2.25) | 9(3.37) | 28(10.49) | 56(20.97) | 168(62.92) | 4.39±0.96 |
|  | Cupping Technique | 6(2.25) | 9(3.37) | 28(10.49) | 53(19.85) | 171(64.04) | 4.40±0.96 |
|  | Direct Moxibustion with Grain - sized Moxa Cones Technique | 23(8.61) | 16(5.99) | 43(16.10) | 52(19.48) | 133(49.81) | 3.96±1.30 |
|  | Direct Moxibustion with Grain - sized Moxa Cones Technique | 23(8.61) | 16(5.99) | 49(18.35) | 47(17.60) | 132(49.44) | 3.93±1.30 |
|  | Suspended Moxibustion Technique | 24(8.99) | 13(4.87) | 51(19.10) | 49(18.35) | 130(48.69) | 3.93±1.30 |
|  | Wax Therapy Technique | 19.0(7.12) | 16.0(5.99) | 36.0(13.48) | 64.0(23.97) | 132.0(49.44) | 4.03±1.23 |
|  | Acupoint Application Technique | 14.0(5.24) | 8.0(3.00) | 31.0(11.61) | 53.0(19.85) | 161.0(60.30) | 4.27±1.11 |
|  | Chinese Medicinal Fumigation and Washing Technique | 18.0(6.74) | 14.0(5.24) | 26.0(9.74) | 60.0(22.47) | 149.0(55.81) | 4.15±1.21 |
|  | Chinese Medicinal Cold Compress Technique | 21.0(7.87) | 13.0(4.87) | 36.0(13.48) | 62.0(23.22) | 135.0(50.56) | 4.04±1.24 |
|  | Chinese Medicinal Hot and Wet Compress Technique | 16.0(5.99) | 16.0(5.99) | 36.0(13.48) | 56.0(20.97) | 143.0(53.56) | 4.10±1.20 |
|  | Chinese Medicinal Topical Application Technique | 20.0(7.49) | 14.0(5.24) | 42.0(15.73) | 55.0(20.60) | 136.0(50.94) | 4.02±1.25 |
|  | Chinese Medicinal Fumigation and Steaming Technique | 21.0(7.87) | 16.0(5.99) | 41.0(15.36) | 60.0(22.47) | 129.0(48.31) | 3.97±1.26 |
|  | Chinese Medicinal Hot Iron Compress Technique | 21.0(7.87) | 14.0(5.24) | 40.0(14.98) | 66.0(24.72) | 126.0(47.19) | 3.98±1.24 |
|  | Chinese Medicinal Iontophoresis Technique | 17.0(6.37) | 13.0(4.87) | 40.0(14.98) | 59.0(22.10) | 138.0(51.69) | 4.08±1.20 |
|  | Acupoint Injection Technique | 29.0(10.86) | 12.0(4.49) | 50.0(18.73) | 58.0(21.72) | 118.0(44.19) | 3.84±1.33 |
|  | Auricular Point Sticking Technique | 13.0(4.87) | 6.0(2.25) | 30.0(11.24) | 54.0(20.22) | 164.0(61.42) | 4.31±1.08 |
|  | Meridian - Acupoint Tuina Technique | 21.0(7.87) | 8.0(3.00) | 33.0(12.36) | 53.0(19.85) | 152.0(56.93) | 4.15±1.23 |
|  | Chinese Medicinal Enema Technique | 23.0(8.61) | 13.0(4.87) | 35.0(13.11) | 54.0(20.22) | 142.0(53.18) | 4.04±1.28 |
| Nursing of Common Syndromes in Traditional Chinese Medicine Internal Medicine | Cough | 31.0(11.61) | 11.0(4.12) | 37.0(13.86) | 66.0(24.72) | 122.0(45.69) | 3.89±1.34 |
|  | Wheezing Syndrome | 31.0(11.61) | 13.0(4.87) | 41.0(15.36) | 65.0(24.34) | 117.0(43.82) | 3.84±1.35 |
|  | Dizziness | 29.0(10.86) | 11.0(4.12) | 29.0(10.86) | 62.0(23.22) | 136.0(50.94) | 3.99±1.33 |
|  | Insomnia | 34.0(12.73) | 12.0(4.49) | 28.0(10.49) | 70.0(26.22) | 123.0(46.07) | 3.88±1.37 |
|  | Palpitation | 31.0(11.61) | 12.0(4.49) | 30.0(11.24) | 68.0(25.47) | 126.0(47.19) | 3.92±1.35 |
|  | Chest Impediment | 35.0(13.11) | 14.0(5.24) | 39.0(14.61) | 68.0(25.47) | 111.0(41.57) | 3.77±1.38 |
|  | Apoplexy | 31.0(11.61) | 12.0(4.49) | 36.0(13.48) | 59.0(22.10) | 129.0(48.31) | 3.91±1.36 |
|  | Stomachache | 31.0(11.61) | 14.0(5.24) | 44.0(16.48) | 66.0(24.72) | 112.0(41.95) | 3.80±1.34 |
|  | Diarrhea | 31.0(11.61) | 15.0(5.62) | 42.0(15.73) | 60.0(22.47) | 119.0(44.57) | 3.83±1.36 |
|  | Constipation | 31.0(11.61) | 11.0(4.12) | 34.0(12.73) | 62.0(23.22) | 129.0(48.31) | 3.93±1.35 |
|  | Edema | 33.0(12.36) | 17.0(6.37) | 42.0(15.73) | 59.0(22.10) | 116.0(43.45) | 3.78±1.38 |
|  | Consumptive Thirst | 31.0(11.61) | 15.0(5.62) | 43.0(16.10) | 60.0(22.47) | 118.0(44.19) | 3.82±1.36 |
|  | Headache | 30.0(11.24) | 16.0(5.99) | 40.0(14.98) | 58.0(21.72) | 123.0(46.07) | 3.85±1.36 |
| Nursing of Common Diseases in Traditional Chinese Medicine Gynecology | Erysipelas | 38.0(14.23) | 18.0(6.74) | 44.0(16.48) | 56.0(20.97) | 111.0(41.57) | 3.69±1.43 |
|  | Acute Mastitis | 39.0(14.61) | 18.0(6.74) | 48.0(17.98) | 60.0(22.47) | 102.0(38.20) | 3.63±1.42 |
|  | Varicose Ulcer | 45.0(16.85) | 22.0(8.24) | 50.0(18.73) | 58.0(21.72) | 92.0(34.46) | 3.49±1.46 |
|  | Hemorrhoids | 44.0(16.48) | 20.0(7.49) | 49.0(18.35) | 61.0(22.85) | 93.0(34.83) | 3.52±1.45 |
|  | Anal Fissure | 44.0(16.48) | 22.0(8.24) | 48.0(17.98) | 57.0(21.35) | 96.0(35.96) | 3.52±1.46 |
|  | Anal Fistula | 46.0(17.23) | 22.0(8.24) | 49.0(18.35) | 59.0(22.10) | 91.0(34.08) | 3.48±1.46 |
|  | Anorectal Abscess | 45.0(16.85) | 22.0(8.24) | 47.0(17.60) | 59.0(22.10) | 94.0(35.21) | 3.51±1.46 |
|  | Uterine Bleeding | 48.0(17.98) | 21.0(7.87) | 47.0(17.60) | 62.0(23.22) | 89.0(33.33) | 3.46±1.47 |
|  | Leukorrheal Disease | 48.0(17.98) | 22.0(8.24) | 43.0(16.10) | 63.0(23.60) | 91.0(34.08) | 3.48±1.48 |
|  | Dysmenorrhea | 43.0(16.10) | 23.0(8.61) | 40.0(14.98) | 67.0(25.09) | 94.0(35.21) | 3.55±1.45 |
|  | Amenorrhea | 48.0(17.98) | 22.0(8.24) | 44.0(16.48) | 65.0(24.34) | 88.0(32.96) | 3.46±1.47 |
|  | Premenstruation, Postmenstruation, Irregular Menstruation | 47.0(17.60) | 24.0(8.99) | 43.0(16.10) | 62.0(23.22) | 91.0(34.08) | 3.47±1.47 |
|  | Infertility | 47.0(17.60) | 23.0(8.61) | 43.0(16.10) | 63.0(23.60) | 91.0(34.08) | 3.48±1.47 |
| Nursing of Common Diseases in Traditional Chinese Medicine Pediatrics | Cough due to Pneumonia | 43.0(16.10) | 19.0(7.12) | 45.0(16.85) | 55.0(20.60) | 105.0(39.33) | 3.60±1.46 |
|  | Asthma | 48.0(17.98) | 18.0(6.74) | 41.0(15.36) | 61.0(22.85) | 99.0(37.08) | 3.54±1.49 |
|  | Anorexia | 50.0(18.73) | 22.0(8.24) | 44.0(16.48) | 59.0(22.10) | 92.0(34.46) | 3.45±1.49 |
|  | Food Stagnation | 49.0(18.35) | 21.0(7.87) | 48.0(17.98) | 61.0(22.85) | 88.0(32.96) | 3.44±1.47 |
|  | Infantile Malnutrition | 51.0(19.10) | 23.0(8.61) | 49.0(18.35) | 61.0(22.85) | 83.0(31.09) | 3.38±1.48 |
|  | Infantile Diarrhea | 52.0(19.48) | 21.0(7.87) | 44.0(16.48) | 61.0(22.85) | 89.0(33.33) | 3.43±1.50 |
|  | Infantile Convulsion | 52.0(19.48) | 21.0(7.87) | 43.0(16.10) | 62.0(23.22) | 89.0(33.33) | 3.43±1.50 |
|  | Enuresis | 53.0(19.85) | 20.0(7.49) | 45.0(16.85) | 60.0(22.47) | 89.0(33.33) | 3.42±1.50 |
| mplementation of TCM Nursing Protocols for Common Emergencies | | 20.0(7.49) | 11.0(4.12) | 39.0(14.61) | 54.0(20.22) | 143.0(53.56) | 4.08±1.23 |
| Implementation of TCM Nursing Protocols for Common Diseases in Internal Medicine | | 30.0(11.24) | 12.0(4.49) | 33.0(12.36) | 52.0(19.48) | 140.0(52.43) | 3.97±1.36 |
| Implementation of TCM Nursing Protocols for Common Diseases in Surgery | | 29.0(10.86) | 15.0(5.62) | 38.0(14.23) | 43.0(16.10) | 142.0(53.18) | 3.95±1.37 |
| Implementation of TCM Nursing Protocols for Common Diseases in Gynecology | | 36.0(13.48) | 20.0(7.49) | 51.0(19.10) | 49.0(18.35) | 111.0(41.57) | 3.67±1.42 |
| Implementation of TCM Nursing Protocols for Common Diseases in Pediatrics | | 37.0(13.86) | 19.0(7.12) | 45.0(16.85) | 54.0(20.22) | 112.0(41.95) | 3.69±1.43 |
