# Supplementary material for: A study on the construction of a nurse refresher training system in traditional Chinese medicine hospitals using the Delphi method
Source: Front Public Health. 2025 Jul 23;13:1618002. doi: 10.3389/fpubh.2025.1618002 (PMC12325172; doi:10.3389/fpubh.2025.1618002)
Supplement: Supplementary file 2 [file Table_2.docx]

Supplementary Table 2 Indicator program for further training system for nursing staff in TCM hospitals

| Indicators | Rating of importance | Coefficient of variation |
| --- | --- | --- |
| 1. Training objective | 4.41±0.70 | 0.16 |
| 1.1 A heart for saving lives and caring for patients with a strong sense of compassion | 4.82±0.40 | 0.08 |
| 1.2 Solid TCM nursing theory | 4.45±0.69 | 0.15 |
| 1.3 A certain degree of practical foundation in TCM nursing skills and further improvement of TCM skills | 4.73±0.47 | 0.10 |
| 1.4 Dialectical care based on patient's mood, diet, rehabilitation, and other nursing care | 4.64±0.50 | 0.11 |
| 1.5 Ability to skillfully apply TCM nursing knowledge and techniques to provide a high level of TCM nursing specialty services to service users | 4.64±0.50 | 0.11 |
| 1.6 Educational ability to provide rehabilitation and health guidance with Chinese medicine characteristics, and to conduct clinical teaching of TCM | 4.36±0.67 | 0.15 |
| 1.7 Demonstrated management skills in TCM nursing management | 3.91±0.83 | 0.21 |
| 1.8 Ability to innovate and conduct research in TCM nursing | 4.00±0.89 | 0.22 |
| 1.9 Ability to popularize TCM nursing science | 4.18±0.75 | 0.18 |
| 2. Admission criteria | 4.59±0.67 | 0.15 |
| 2.1 Possess a professional qualification as a Registered Nurse | 4.82±0.40 | 0.08 |
| 2.2 Graduated from TCM nursing program, graduated from Western medicine nursing program, and attended the training course of Western learning Chinese or systematically received TCM nursing knowledge and skills training | 4.36±0.81 | 0.19 |
| 3. Training method | 4.41±0.75 | 0.17 |
| 3.1 Forms of instruction | 4.33±0.81 | 0.19 |
| 3.1.1 Intensive course | 4.64±0.67 | 0.15 |
| 3.1.2 Section team teaching | 4.55±0.82 | 0.18 |
| 3.1.3 Online course | 3.73±0.79 | 0.21 |
| 3.1.4 Study by oneself | 4.27±0.79 | 0.18 |
| 3.2 Methods of instruction | 4.53±0.71 | 0.16 |
| 3.2.1 Classroom teaching | 4.73±0.47 | 0.10 |
| 3.2.2 Panel discussion | 4.64±0.67 | 0.15 |
| 3.2.3 Operational room inspection | 4.64±0.50 | 0.11 |
| 3.2.4 Operating instructions | 4.73±0.47 | 0.10 |
| 3.2.5 Workshop | 3.82±1.08 | 0.28 |
| 3.2.6 Case care | 4.45±0.69 | 0.15 |
| 3.2.7 Case discussion | 4.55±0.52 | 0.11 |
| 3.2.8 Special lecture | 4.18±0.75 | 0.18 |
| 3.3 Training hours | 4.77±0.43 | 0.09 |
| 3.3.1 Theoretical hours | 4.73±0.47 | 0.10 |
| 3.3.2 Practical hours | 4.82±0.40 | 0.08 |
| 3.4 Practice places | 4.73±0.55 | 0.12 |
| 3.4.1 Integrated Chinese and Western medicine ward | 4.82±0.40 | 0.08 |
| 3.4.2 TCM nursing clinic | 4.64±0.67 | 0.15 |
| 4. Nursing professionalism | 4.67±0.54 | 0.12 |
| 4.1 Basic contents of professional ethics for nurses | 4.82±0.40 | 0.08 |
| 4.2 Nurses professional image and etiquette norms | 4.55±0.69 | 0.15 |
| 4.3 Essential elements of nursing ethics | 4.64±0.50 | 0.11 |
| 5. Laws, regulations and rules | 4.68±0.48 | 0.10 |
| 5.1 Nurses ordinance | 4.64±0.50 | 0.11 |
| 5.2 Nursing regulations and procedures | 4.73±0.47 | 0.10 |
| 6. Basic TCM course | 4.29±0.72 | 0.17 |
| 6.1 Basic theory of TCM | 4.73±0.47 | 0.10 |
| 6.2 TCM Diagnosis | 4.18±0.75 | 0.18 |
| 6.2.1 The contents and methods of the four diagnostic methods: looking, smelling, questioning, and cutting. | 4.45±0.52 | 0.12 |
| 6.2.2 Pattern-syndrome identification based on the eight principles (TCM) | 4.55±0.52 | 0.11 |
| 6.2.3 Identification of internal organs | 4.27±0.65 | 0.15 |
| 6.2.4 Pattern-syndrome identification | 3.91±0.83 | 0.21 |
| 6.3 TCM and health sciences | 4.45±0.52 | 0.12 |
| 6.3.1 Basic principles of health maintenance and common health maintenance methods | 4.36±0.67 | 0.15 |
| 6.3.2 Health and wellness methods | 4.27±0.79 | 0.18 |
| 7. Basic TCM nursing course | 4.48±0.76 | 0.17 |
| 7.1 Fundamentals of TCM nursing | 4.82±0.40 | 0.08 |
| 7.1.1 Basic features of TCM nursing | 4.64±0.50 | 0.11 |
| 7.1.3 General nursing care (condition observation, living and living care, etc.) | 4.64±0.50 | 0.11 |
| 7.1.4 Evidence-based care (identification of the eight principles, identification of internal organs, identification of care, identification of medicine, identification of diet, identification of education, identification of nutrition, etc.) | 4.73±0.47 | 0.10 |
| 7.2 TCM and food therapy | 4.55±0.52 | 0.11 |
| 7.3 Basic knowledge of meridians and acupuncture points | 4.36±0.81 | 0.19 |
| 7.3.1 Concept and composition of meridians | 4.27±0.90 | 0.21 |
| 7.3.2 Names, distribution, and routes of the twelve meridians | 4.36±0.81 | 0.19 |
| 7.3.3 Concepts and physiological functions of the eight meridians | 4.00±0.89 | 0.22 |
| 7.3.4 Basic knowledge of acupuncture points | 4.55±0.69 | 0.15 |
| 7.3.5 Commonly used acupuncture (positioning, efficacy, and main treatment, precautions for operation, methods of clinical application, principles of selecting acupuncture) | 4.82±0.40 | 0.08 |
| 7.3.6 Acupuncture points for common diseases | 4.82±0.40 | 0.08 |
| 8. Characteristic TCM nursing techniques | 4.58±0.61 | 0.13 |
| 8.1 TCM nursing techniques | 4.58±0.61 | 0.13 |
| 8.1.1 Fire cupping | 4.73±0.47 | 0.10 |
| 8.1.2 Gua sha | 4.73±0.47 | 0.10 |
| 8.1.3 Ear point pressure | 4.64±0.50 | 0.11 |
| 8.1.4 Acupuncture point paste | 4.73±0.47 | 0.10 |
| 8.1.5 Meridian point massage | 4.55±0.69 | 0.15 |
| 8.1.6 Acupuncture point injection | 4.55±0.69 | 0.15 |
| 8.1.7 Sandwiched moxibustion | 4.73±0.47 | 0.10 |
| 8.1.8 Wheat grain moxibustion | 4.45±0.82 | 0.18 |
| 8.1.9 Suspended moxibustion | 4.55±0.69 | 0.15 |
| 8.1.10 Chinese herb hot compresses | 4.55±0.69 | 0.15 |
| 8.1.11 Cold compresses with Chinese herbs | 4.55±0.69 | 0.15 |
| 8.1.12 Damp-Heat Chinese herb compresses | 4.73±0.47 | 0.10 |
| 8.1.13 Chinese herbal fumigation | 4.64±0.50 | 0.11 |
| 8.1.14 Chinese herb application | 4.55±0.52 | 0.11 |
| 8.1.15 Chinese herb enema | 4.55±0.52 | 0.11 |
| 8.1.16 Chinese herb collapse | 4.64±0.67 | 0.15 |
| 8.1.17 Chinese herb poultice | 4.45±0.82 | 0.18 |
| 8.1.18 Chinese herb hot dying pack | 4.55±0.69 | 0.15 |
| 8.1.19 TCM five elements music therapy | 4.27±0.79 | 0.18 |
| 8.2 Nutritional rehabilitation | 4.55±0.69 | 0.15 |
| 8.2.1 Functional training for health maintenance and rehabilitation of common diseases | 4.55±0.69 | 0.15 |
| 9. TCM nursing professional programs | 4.65±0.56 | 0.12 |
| 9.1 TCM nursing for common medical conditions in internal medicine | 4.82±0.40 | 0.08 |
| 9.1.1 Cough | 4.73±0.47 | 0.10 |
| 9.1.2 Asthma | 4.64±0.50 | 0.11 |
| 9.1.3 Dizziness | 4.64±0.50 | 0.11 |
| 9.1.4 Insomnia | 4.64±0.50 | 0.11 |
| 9.1.5 Palpitation | 4.73±0.47 | 0.10 |
| 9.1.6 Chest paralysis | 4.73±0.47 | 0.10 |
| 9.1.7 Heart failure | 4.73±0.47 | 0.10 |
| 9.1.8 Stroke | 4.73±0.47 | 0.10 |
| 9.1.9 Stomachache | 4.55±0.69 | 0.15 |
| 9.1.10 Diarrhoea | 4.55±0.69 | 0.15 |
| 9.1.11 Constipation | 4.73±0.47 | 0.10 |
| 9.1.12 Oedema | 4.64±0.67 | 0.15 |
| 9.1.13 Thirst | 4.73±0.47 | 0.10 |
| 9.1.14 Headache | 4.64±0.50 | 0.11 |
| 9.1.15 Rheumatic paralysis | 4.55±0.69 | 0.15 |
| 9.1.16 Cancer | 4.73±0.47 | 0.10 |
| 9.2 TCM nursing of common surgical conditions | 4.73±0.47 | 0.10 |
| 9.2.1 Deng poison | 4.45±0.82 | 0.18 |
| 9.2.2 Carbuncles | 4.64±0.67 | 0.15 |
| 9.2.3 Polycarbonates | 4.64±0.67 | 0.15 |
| 9.2.4 Haemorrhoids | 4.55±0.69 | 0.15 |
| 9.2.5 Anal fissure | 4.55±0.69 | 0.15 |
| 9.2.6 Anal fistula | 4.64±0.67 | 0.15 |
| 9.2.7 Anal carbuncle | 4.64±0.67 | 0.15 |
| 9.2.8 Guan Ge (Intestinal Obstruction) | 4.73±0.47 | 0.10 |
| 9.2.9 Paralysis of the neck | 4.73±0.47 | 0.10 |
| 9.2.10 Knee paralysis | 4.73±0.47 | 0.10 |
| 9.2.11 Fracture | 4.64±0.50 | 0.11 |
| 9.2.12 Lumbago (Lumbar Disc Herniation) | 4.73±0.47 | 0.10 |
| 9.3 TCM nursing for common gynecological conditions | 4.73±0.47 | 0.10 |
| 9.3.1 Metrorrhagia | 4.73±0.47 | 0.10 |
| 9.3.2 Dysmenorrhoea | 4.55±0.69 | 0.15 |
| 9.3.3 Belt Disorder | 4.27±1.01 | 0.24 |
| 9.4 Paediatric nursing in TCM nursing | 4.55±0.69 | 0.15 |
| 9.4.1 Colds and flu | 4.64±0.50 | 0.11 |
| 9.4.2 Diarrhoea | 4.64±0.50 | 0.11 |
| 9.4.3 Pneumonia and cough | 4.73±0.47 | 0.10 |
| 10. TCM nursing scheme | 4.56±0.60 | 0.13 |
| 10.1 Implementation of TCM nursing schemes for common acute diseases | 4.45±0.69 | 0.15 |
| 10.2 Implementation of TCM Nursing schemes for Common Internal Medicine Diseases | 4.64±0.50 | 0.11 |
| 10.3 Implementation of TCM nursing schemes for common surgical diseases | 4.64±0.50 | 0.11 |
| 10.4 Implementation of TCM nursing schemes for common gynecological diseases | 4.55±0.69 | 0.15 |
| 10.5 Implementation of TCM nursing schemes for common pediatric conditions | 4.55±0.69 | 0.15 |
| 11. Nursing management capacity | 4.34±0.64 | 0.15 |
| 11.1 Operation and management of integrated Chinese and Western medicine nursing wards | 4.36±0.81 | 0.19 |
| 11.2 Response to emergencies in TCM nursing wards | 4.18±0.75 | 0.18 |
| 11.3 The opening and operation of TCM nursing outpatient clinic | 4.36±0.50 | 0.12 |
| 11.4 Response to emergencies in TCM nursing outpatient clinics | 4.45±0.52 | 0.12 |
| 12. Nursing education capacity | 4.68±0.57 | 0.12 |
| 12.1 Health science in TCM nursing | 4.73±0.47 | 0.10 |
| 12.2 Nursing teaching competence | 4.64±0.67 | 0.15 |
| 13. Nursing research capacity | 4.41±0.59 | 0.13 |
| 13.1 Application of clinical practice guidelines in TCM hospitals | 4.45±0.52 | 0.12 |
| 13.2 Fundamentals of research in TCM nursing | 4.36±0.67 | 0.15 |
| 14. Professional development capacity | 4.36±0.67 | 0.15 |
| 14.1 Construction and prospect of TCM nursing discipline | 4.36±0.67 | 0.15 |
| 15. Assessment indicators | 4.61±0.70 | 0.15 |
| 15.1. TCM nursing theory assessment (complete the assessment in monthly stages) | 4.55±0.82 | 0.18 |
| 15.2. TCM nursing skills assessment | 4.73±0.47 | 0.10 |
| 15.3. Five case reports (choose one) | 4.55±0.82 | 0.18 |
